# Draft Genome Sequence of a Serratia marcescens Strain Isolated from the Pitcher Fluids of a *Sarracenia* Pitcher Plant

**DOI:** 10.1128/MRA.01216-19

**Published:** 2020-01-09

**Authors:** Shumeng Zhang, Srinivasa R. Chaluvadi, Jeffrey L. Bennetzen

**Affiliations:** a Institute of Bioinformatics, University of Georgia, Athens, Georgia, USA; b Department of Genetics, University of Georgia, Athens, Georgia, USA; University of Arizona

## Abstract

The genome of a Serratia marcescens strain (C7) that was found in the pitcher fluids of a *Sarracenia rosea* pitcher plant was sequenced using the Illumina platform. A 5,543,750-bp genome assembly was obtained. A total of 6,278 coding sequences are predicted from this assembly.

## ANNOUNCEMENT

Serratia marcescens is a species of facultatively anaerobic bacteria that stains as Gram-negative rods (1). The genus *Serratia* is in the family *Enterobacteriaceae* (1). S. marcescens strains were previously isolated from soil (2) and plant tissue (3). Some strains can promote enhanced plant growth and confer abiotic and/or biotic stress tolerance (3–5). S. marcescens has also been isolated from humans (6). Some S. marcescens strains cause various diseases, such as meningitis (7), urinary tract infections, and wound infections (8). S. marcescens has also been found in infections of other animals, including some insects (9).

The strain investigated in this study (C7) was isolated from the pitcher fluids of Sarracenia rosea pitcher plants from Splinter Hill Bog, AL (collected in March 2012). The strain was isolated from pitcher fluids by plating on medium used for the culture of Pyrococcus furiosus (10); single colonies were serially streaked on the same medium three times, and then an individual colony was picked into nutrient broth (11) for growth and eventual storage at –80°C in 25% glycerol. DNA was extracted using the Quick-DNA fungal/bacterial miniprep kit (product number D6005; Zymo Research). A paired-end library was prepared using the Nextera XT DNA library preparation kit v2 (product number FC-131-1002; Illumina), with an average insert size of ∼400 bp, for Illumina MiSeq sequencing. Of 17,917,430 Illumina raw reads, we retained 5,643,558 reads after quality filtering with Trimmomatic v0.36 (12). Then, FastQC v0.11.4 was employed to check the quality of the trimmed reads (13). SOAPdenovo2 r240 (parameter K, 83) was used to assemble the reads into contigs (14). Contigs with lengths of <400 bp were discarded. In total, 1,306 contigs were selected to be ordered in Mauve (15), using Serratia marcescens strain FDAARGOS_65 (GenBank accession number NZ_CP026050) as the reference genome. The draft genome was annotated using the online RAST server (16). Default parameters were used for all software tools, unless otherwise noted.

**FIG 1:**
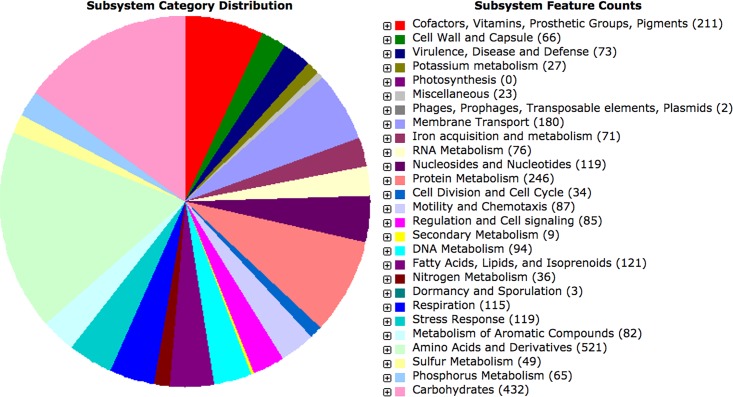
Subsystem distribution based on RAST SEED analysis. The pie chart organizes the presented subsystems by cellular process, and the number of protein-coding genes (in parentheses) that are predicted to be involved in that cellular process are indicated.

The final genome assembly was 5,543,750 bp, with a GC content of 58.1%. The genome coverage was ∼300×, the *N*_50_ was 22,377 bp, and the *L*_50_ was 80. There were 6,278 coding sequences predicted in total, consisting of 6,189 protein-coding sequences and 89 RNA genes. Only 34.2% of the protein-coding sequences (2,122 genes) for this genome could be grouped into the functional subsystems of the RAST SEED server (17). The subsystem category distribution is shown in Fig. 1.

CGE ResFinder (18) was used to predict antibiotic resistance genes. The genome assembly was predicted to contain the beta-lactam resistance gene *bla*_ACT-6_ and the aminoglycoside resistance gene *aac(6′)-Ic*. CGE PathogenFinder (19) predicted that the strain had a 77.2% probability of being a human pathogen. The average nucleotide identities (20) determined by comparison with the two most closely related bacterial strains (identified with BLASTn) in the NCBI database, Serratia marcescens strain AS1 (GenBank accession number CP010584) and Serratia marcescens strain UMH5 (GenBank accession number CP018917), were both ∼94.2%; the average amino acid identities were 96.1% and 96.0%, respectively. When PATRIC (21, 22) was used for comparison with these two other S. marcescens strains (both from clinical samples), the pitcher S. marcescens strain was determined to have a unique fluorobenzoate degradation pathway and an increase in the number of genes involved in sulfur metabolism. This comparison also demonstrated that the pitcher strain lacked carbamate kinase (EC 2.7.2.2) but had two more glutamate ammonia ligase (EC 6.3.1.2) and two more glutamate synthase (NADPH) (EC 1.4.1.13) genes involved in nitrogen metabolism.

### Data availability.

This whole-genome shotgun project has been deposited in GenBank under accession number NZ_QPFX00000000. The raw read SRA accession number is SRX6867789. The version described in this paper is the first version. The BioProject accession number is PRJNA481376. The BioSample accession number is SAMN09666492.
